# Breakdown of Autonomously Functioning Thyroid Nodule Accompanied by Acromegaly After Octreotide Treatment

**DOI:** 10.3389/fendo.2019.00131

**Published:** 2019-03-01

**Authors:** Hiroshi Nomoto, Hiraku Kameda, Akinobu Nakamura, Kazuhisa Tsuchida, So Nagai, Tatsuya Atsumi, Hideaki Miyoshi

**Affiliations:** ^1^Department of Rheumatology, Endocrinology and Nephrology, Faculty of Medicine and Graduate School of Medicine, Hokkaido University, Sapporo, Japan; ^2^Division of Diabetes and Obesity, Faculty of Medicine and Graduate School of Medicine, Hokkaido University, Sapporo, Japan

**Keywords:** autonomously functioning thyroid nodule, acromegaly, somatostatin analog, pituitary adenoma, thyrotoxicosis

## Abstract

Patients with acromegaly are at increased risk of developing certain tumors, including goiter and thyroid nodules, and occasionally autonomous thyroid nodules. A 53-year-old woman presented at our hospital with untreated acromegaly. She had typical physical features of acromegaly with pituitary adenoma, and thyrotoxicosis with thyroid-stimulating hormone suppression was also confirmed. Thyroid ultrasonography and scintigraphy showed an autonomously functioning thyroid nodule on her right lobe. Because her thyrotoxicosis was mild, she was initially treated with octreotide for acromegaly. However, 1 month after octreotide administration, she developed neck pain and fever with transient thyrotoxicosis. The blood flow around the nodule then decreased and the excess trapping of isotope detected by scintigraphy was reduced, followed by normalization of insulin-like growth factor-1 levels and thyroid function. This case suggests that octreotide may have unexpected effects on autonomous thyroid nodules. However, further studies are needed to determine the clinical course of autonomously functioning thyroid nodules, including thyroid function and tumor manifestations, during octreotide therapy.

## Introduction

Patients with acromegaly are known to be at increased risk of developing certain tumors as a result of long-term excessive exposure to growth hormone (GH) and insulin-like growth factor (IGF)-1, which stimulate cell proliferation and have an anti-apoptotic effect (1, 2). It is therefore important to detect such complications. Thyroid nodules and goiters are particularly frequent in patients with acromegaly compared with non-disease controls (3). Most of these are adenomatous goiters (4), but autonomous thyroid nodules can also occur in up to 10% of cases (5). Autonomously functioning thyroid nodules (AFTNs) are thyroid-stimulating hormone (TSH)-independent, thyroid hormone-secreting nodules against a background of a TSH-responsive thyroid, and appear as nodules that have excess trapping of isotope compare to the surrounding normal thyroid tissue in technetium-99 m pertechnetate scintigraphy. Treatment of AFTNs generally involves surgery, radioactive iodine therapy, or percutaneous ethanol injection (6, 7). Although the somatostatin receptor (SSTR) ligand, octreotide, is known to be a useful treatment option for acromegaly (8), its effects on AFTNs remain unclear.

We herein provide the first report of a patient with acromegaly and AFTN who showed destruction of AFTN followed by normalization of hyperthyroidism after treatment with octreotide.

## Background

**Figure 1:**
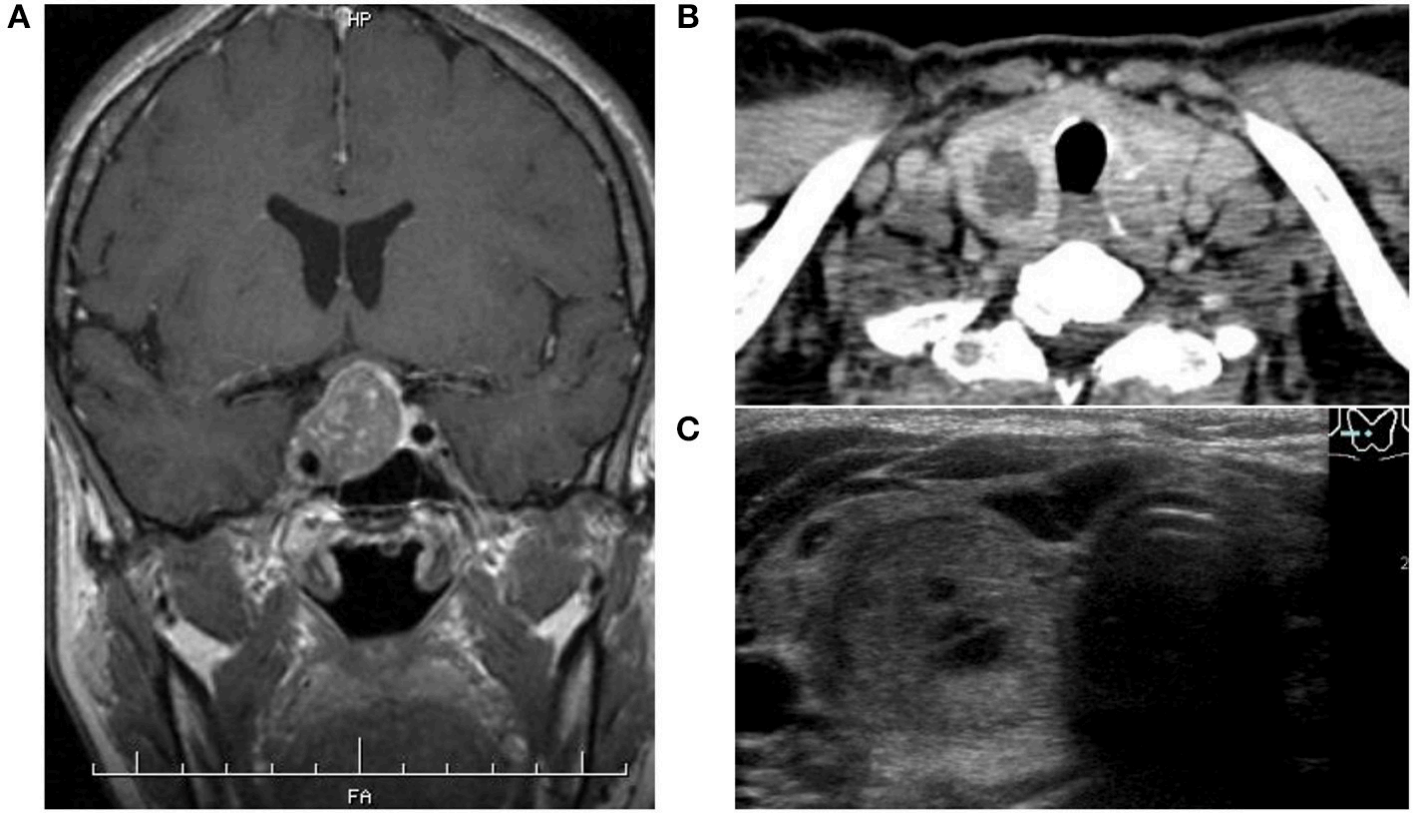
**(A)** Magnetic resonance imaging showed a pituitary tumor demonstrated less enhancement than the normal pituitary gland after intravenous contrast administration with invasion of the right cavernous sinus. **(B,C)** Thyroid enhanced computed tomography and ultrasonography showed several nodules inside the thyroid gland, of which the largest located at the bottom of the right lobe contained a cystic component and high vascularity.

**Figure 2:**
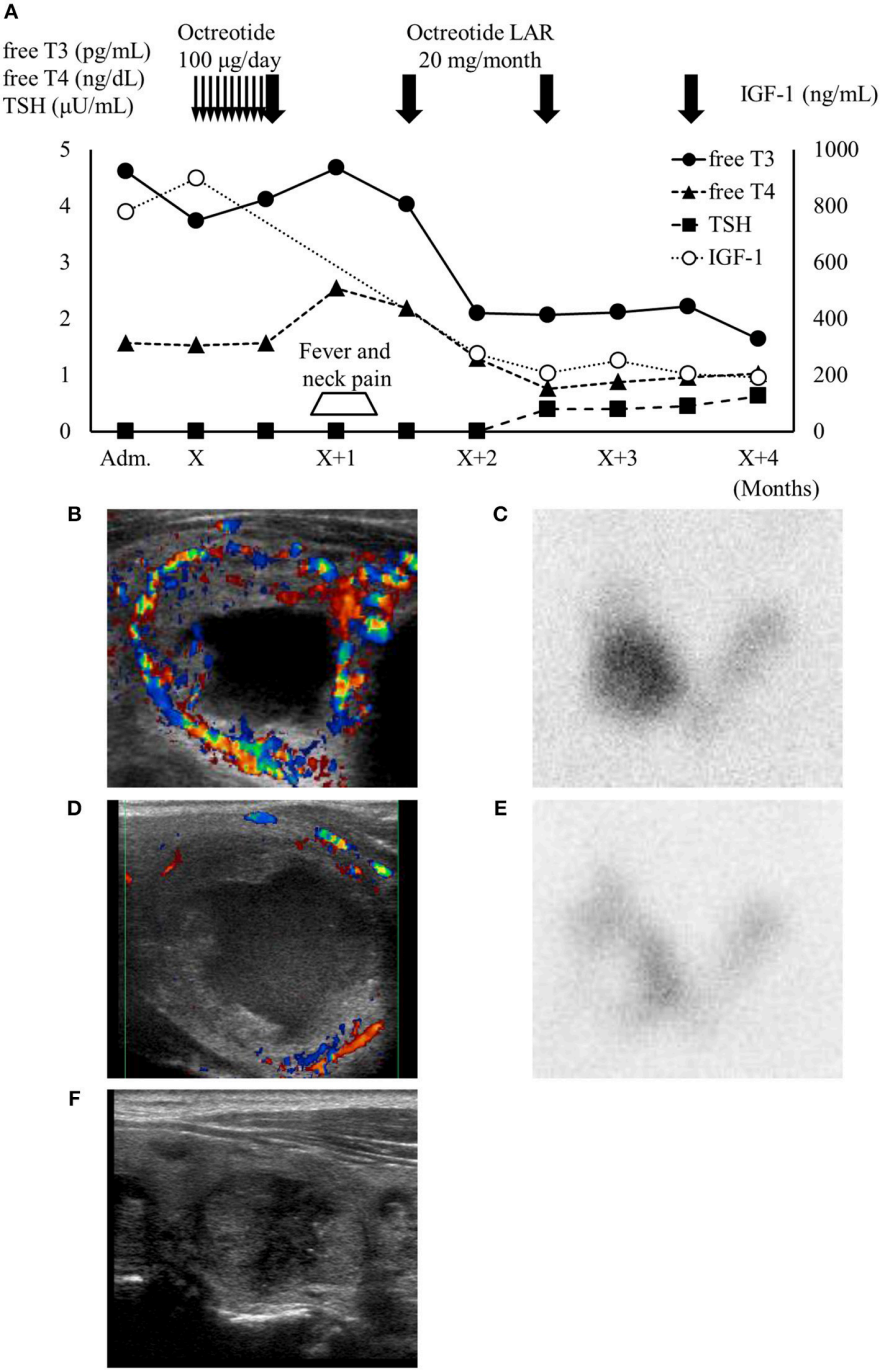
**(A)** High IGF-1 level and thyrotoxicosis with TSH suppression were confirmed on admission. One month after octreotide treatment, the patient developed neck pain and fever accompanied by transient thyroid hormone elevation. IGF-1 levels and thyroid function subsequently normalized under octreotide treatment. Thin arrows, daily octreotide injection (100 μg/day); bold arrows, monthly octreotide-LAR injection (20 mg/month). **(B,C)** Thyroid ultrasonography (US) showed the largest thyroid nodule in the right lobe (34.4 × 25.3 × 23.3 mm) with high vascularity and a cystic region. This nodule showed as excess trapping of isotope in technetium-99 m pertechnetate scintigraphy against a low background of the thyroid. **(D,E)** One month after octreotide long-acting release treatment, the patient developed right cervical pain and temporary worsening of thyrotoxicosis. At that point, the blood flow surrounding the nodule decreased and the accumulation shown by scintigraphy was reduced. **(F)** Four months after octreotide treatment, thyroid US showed that the autonomous thyroid nodule had shrunk (20.3 × 13.8 × 12.8 mm) and most of the cystic lesion had been reduced. Adm., administration day; X, first day of octreotide treatment; LAR, long-acting release.

A 53-year-old woman with an unusual facial configuration noted 9 years earlier who also had enlarged fingers was admitted to our hospital with suspected acromegaly. She had a history of colon polyps, but her family history was negative for both pituitary and thyroid diseases. She had never smoked and did not consume alcohol regularly, and was receiving no medications and supplements. On admission, her height was 154 cm, her body weight was 54.4 kg, and her body mass index was 22.9 kg/m^2^. Her thyroid gland was slightly enlarged, and a hard non-painful 30 mm nodule was palpable at the bottom of the right lobe. She demonstrated the typical physical signs of acromegaly, including somatic enlargement, jaw overgrowth, and excessive sweating, but there was no skin rash and no signs of any other endocrinopathies. She also showed tachycardia (103 beats per min). Fasting morning blood analysis showed high levels of GH (11.2 ng/mL) and IGF-1 (781 ng/mL), and thyrotoxicosis accompanied by suppression of TSH (Table 1). Additional blood analysis confirmed that thyroglobulin antibody, anti-thyroid peroxidase antibody, TSH receptor antibody, and thyroid stimulating antibody were all negative (Table 1). Her GH levels were not suppressed during a 75 g oral glucose tolerance test. Magnetic resonance imaging revealed a pituitary tumor (measuring 25 × 25 × 23 mm) with invasion of the right cavernous sinus (Knosp grade III) (Figure 1A). Based on her physical features, pituitary tumor, and glucose tolerance test result, the patient was diagnosed with acromegaly. Thyroid ultrasonography and computed tomography showed multiple nodules inside the thyroid gland, of which the largest nodule located in the lower right lobe was partially cystic with a rich blood supply (Figures 1B,C, 2B). She was therefore also diagnosed with an AFTN based on the results of technetium-99 m pertechnetate scintigraphy (Figure 2C). Whole-body examination and laboratory findings suggested no tumors, including in the endocrine organs, other than the pituitary, and thyroid gland.

**Table 1:** Laboratory tests on admission.

**Variable**	**Reference range**	**Value**
GH (ng/mL)	<2.1	11.2
IGF-1 (ng/mL)	77–212	781
LH (mIU/mL)	14.4–62.2	16.6
FSH (mIU/mL)	36.6–168.8	43.9
Estradiol (pg/mL)	<10.0	<10.0
TSH (μU/mL)	0.38–4.31	<0.01
fT3 (pg/mL)	2.1–3.8	4.44
fT4 (ng/dL)	0.82–1.63	1.80
ACTH (pg/mL)	7.2–63.3	45.4
Cortisol (μg/dL)	4.0–23.3	6.8
PRL (ng/mL)	4.5–28.5	43.0
intact-PTH (pg/mL)	10–65	43
Anti-thyroglobulin antibody (IU/mL)	<28	<28
Anti-thyroid peroxidase antibody (IU/mL)	<16	<16
Thyroid stimulating antibody (%)	<120	<120
TSH receptor antibody (IU/L)	<2.0	<2.0

*GH, growth hormone; IGF-1, insulin-like growth factor-1; LH, luteinizing hormone; FSH, follicle-stimulating hormone; TSH, thyroid-stimulating hormone; ACTH, adrenocorticotropic hormone; PRL, prolactin; PTH, parathyroid hormone*.

Because cavernous sinus invasion of the pituitary tumor was suspected, we administrated daily octreotide injections (100 μg/day) for 2 weeks followed by monthly octreotide long-acting release (LAR) (20 mg/month), leading to effective suppression of excessive GH. However, she developed fever and right neck pain 1 month after octreotide administration, accompanied by a transient elevation of thyroid hormones (Figure 2A). At that point, thyroid ultrasonography showed debris-like material inside the cystic lesion and reduced blood flow compared with the previous scan (Figure 2D). The excess trapping of isotope detected by scintigraphy had also decreased (Figure 2E). There were no obvious changes in the other thyroid nodules. Her fever and neck pain were treatable with non-steroidal anti-inflammatory drug and antibiotics, and we therefore continued octreotide-LAR. Her pituitary tumor shrank from 25 × 25 × 23 mm to 18 × 21 × 21 mm as well as AFTN (Figure 2F), and her free T3, T4, and IGF-1 levels normalized after 4 months octreotide treatment. The patient has never relapsed into thyrotoxicosis to date.

## Discussion

To the best of our knowledge, this is the first reported case of a patient with acromegaly in whom an automaticity of an AFTN was completely suppressed by octreotide treatment. SSTR ligands including octreotide and lanreotide, are well-established to be useful for managing acromegaly both in controlling hormone levels and tumor shrinkage (9–12). The SSTR ligand, octreotide, acts mainly on SSTR2-expressing GH-producing pituitary adenomas (9–11, 13), and has also been reported to be beneficial in some SSTR2-positive hormone-excess disorders such as insulinoma (14, 15) and gastroenteropancreatic neuroendocrine tumors (16, 17). However, there is currently no direct evidence for the efficacy of octreotide in AFTN. There have been inconsistent reports regarding SSTR2 expression patterns in autonomous thyroid nodules (18, 19).

The current patient showed AFTN destruction with release of excess thyroid hormone after octreotide therapy. It is possible that this patient's AFTN might have expressed SSTR2 and thus responded to octreotide LAR. Notably, the AFTN but not the other thyroid nodules shrank after octreotide treatment. Previous ultrasonographic study also showed that octreotide therapy for active acromegaly resulted in a reduction of goiter size (20). In addition, an *in vitro* study using a rat thyroid follicular cell line showed that serum from patients with acromegaly increased DNA synthesis compared with serum from normal subjects, while DNA synthesis was decreased by serum from hypothyroid patients (21). These results suggest the possibility that reduction of IGF-1 itself caused by octreotide treatment exerted inhibitory effects on AFTN via suppression of incremental DNA synthesis. Another study reported that both GH and IGF-1 were assumed to affect thyroid growth because thyroid cells synthesize IGF-1 under the control of GH, and also express IGF-1 receptor (22). However, octreotide notably failed to reduce nodule size in subjects with nodule goiters (20). These facts imply that octreotide treatment seemed to affect only AFTN in our case of patient; however, the patient did not undergo resection of the AFTN and we were therefore unable to assess its SSTR expression status.

It is more likely that treatment of the patient's acromegaly might have changed the blood supply to the AFTN, given that the intra-thyroid blood flow is known to be increased in patients with acromegaly and can be normalized by octreotide treatment (23). In addition, somatostatin analogs inhibit angiogenesis via the downregulation of nitric oxide and vascular endothelial growth factor, eliciting anti-tumor effects (17). In fact, the vascularity around the AFTN was obviously reduced after octreotide treatment in our patient. Overall, it is possible that a lack of vascular flow to the already partially cystic nodule, perhaps due to a reduction in GH/IGF-1 by octreotide, caused loss of function of the AFTN through hypoxia and progressive hemorrhagic/cystic destruction of the nodule. Periodic ultrasound assessment confirmed shrinkage of this nodule, but we were unable to confirm the changes histologically.

Regarding thyroid function, excessive GH stimulates peripheral conversion from T4 to T3, which effect is reversed by octreotide (24). Indeed, the T3/T4 ratio appeared to be ameliorated after octreotide LAR administration in the current patient. Moreover, octreotide has been shown to suppress both GH and TSH secretion in patients with acromegaly, with no significant effect on thyroid function (8). Octreotide treatment appeared to trigger destructive inflammation of the AFTN in the present case, accompanied by transient worsening of her hyperthyroidism, and neck pain, followed by complete resolution of her hyperthyroidism. The reduction in the accumulation of technetium-99 m pertechnetate on the nodule suggested that cystic degeneration of the nodule and tissue destruction resulted in death of the autonomous tissue. Although we were unable to perform a pituitary stimulation test because of the large size of the pituitary tumor, her TSH levels recovered to the normal range during the patient's clinical course. This suggests that the TSH suppression was due to excessive thyroid hormone secretion by the AFTN, rather than impaired TSH secretion due to the pituitary tumor.

The current patient suffered from both acromegaly and hyperthyroidism. Although there was a possibility of multiple endocrinopathies including McCune-Albright syndrome, there were no obvious physical or laboratory signs of other endocrinopathies. The coexistence of functioning thyroid nodules and acromegaly has been reported previously (5). AFTNs may develop as a result of somatic gene mutations in the TSH receptor (25) or in the alpha subunit of the stimulatory G protein (26), which is also known to cause GH-producing pituitary adenomas (27). Both GH and IGF-1 are assumed to affect thyroid growth (22), however, whether such GH/IGF-1 stimulation could induce AFTN or if these endocrine disorders share a common background remains unclear.

## Concluding Remarks

In conclusion, we report a patient with AFTN accompanied by acromegaly who was effectively treated with octreotide. This case suggests that octreotide may have some unexpected effects on AFTN, followed by normalization of not only IGF-1, but also thyroid function. However, further studies are needed to determine the clinical course of AFTN, including thyroid function and tumor manifestations, during octreotide therapy.

## Ethics Statement

The need for ethical approval for this case report was waived by the ethical approval or institutional review board of Hokkaido University Hospital, based upon their policy to review all interventional and observational studies, except for case reports. The patient provided written informed consent for the publication of her clinical data. The presented data have been anonymized and the risk of identification is minimal.

## Author Contributions

HN contributed to patient care, wrote the manuscript, and contributed to discussion. HK, KT, and SN contributed to patient care and discussion of diagnosis, and revision of the manuscript. AN, TA, and HM contributed to discussion and revision of the manuscript.

### Conflict of Interest Statement

The authors declare that the research was conducted in the absence of any commercial or financial relationships that could be construed as a potential conflict of interest.

## Abbreviations

AFTN: autonomously functioning thyroid nodules
GH: growth hormone
IGF-1: insulin-like growth factor-1
LAR: long-acting release
SSTR: somatostatin receptor
TSH: thyroid-stimulating hormone.

## References

[1] Jenkins PJ Besser M. Clinical perspective: acromegaly and cancer: a problem. J Clin Endocrinol Metab. (2001) 86:2935–41. 10.1210/jcem.86.7.7634 11443146

[2] Baixeras E Jeay S Kelly PA Postel-Vinay MC. The proliferative and antiapoptotic actions of growth hormone and insulin-like growth factor-1 are mediated through distinct signaling pathways in the Pro-B Ba/F3 cell line. Endocrinology. (2001) 142:2968–77. 10.1210/endo.142.7.8242 11416018

[3] Reverter JL Fajardo C Resmini E Salinas I Mora M Llatjos M. Benign and malignant nodular thyroid disease in acromegaly. Is a routine thyroid ultrasound evaluation advisable? PLoS ONE. (2014) 9:e104174. 10.1371/journal.pone.0104174 25127456 PMC4134205

[4] Kasagi K Shimatsu A Miyamoto S Misaki T Sakahara H Konishi J. Goiter associated with acromegaly: sonographic and scintigraphic findings of the thyroid gland. Thyroid. (1999) 9:791–6. 10.1089/thy.1999.9.791 10482372

[5] Wuster C Steger G Schmelzle A Gottswinter J Minne HW Ziegler R. Increased incidence of euthyroid and hyperthyroid goiters independently of thyrotropin in patients with acromegaly. Horm Metab Res. (1991) 23:131–4. 10.1055/s-2007-1003632 1907592

[6] Hamburger JI. The autonomously functioning thyroid nodule: goetsch's disease. Endocr Rev. (1987) 8:439–47. 10.1210/edrv-8-4-439 3319531

[7] Livraghi T Paracchi A Ferrari C Reschini E Macchi RM Bonifacino A. Treatment of autonomous thyroid nodules with percutaneous ethanol injection: 4-year experience. Radiology. (1994) 190:529–33. 10.1148/radiology.190.2.8284411 8284411

[8] Barkan AL Kelch RP Hopwood NJ Beitins IZ. Treatment of acromegaly with the long-acting somatostatin analog SMS 201-995. J Clin Endocrinol Metab. (1988) 66:16–23. 10.1210/jcem-66-1-16 2891720

[9] Murray RD Melmed S. A critical analysis of clinically available somatostatin analog formulations for therapy of acromegaly. J Clin Endocrinol Metab. (2008) 93:2957–68. 10.1210/jc.2008-0027 18477663

[10] Cozzi R Attanasio R. Octreotide long-acting repeatable for acromegaly. Expert Rev Clin Pharmacol. (2012) 5:125–43. 10.1586/ecp.12.4 22390555

[11] Giustina A Karamouzis I Patelli I Mazziotti G. Octreotide for acromegaly treatment: a reappraisal. Expert Opin Pharmacother. (2013) 14:2433–47. 10.1517/14656566.2013.847090 24124691

[12] Katznelson L Laws ER Jr Melmed S Molitch ME Murad MH Utz A. Acromegaly: an endocrine society clinical practice guideline. J Clin Endocrinol Metab. (2014) 99:3933–51. 10.1210/jc.2014-2700 25356808

[13] Gillis JC Noble S Goa KL. Octreotide long-acting release (LAR). A review of its pharmacological properties and therapeutic use in the management of acromegaly. Drugs. (1997) 53:681–99. 10.2165/00003495-199753040-00009 9098666

[14] Nakamura A Mitsuhashi T Takano Y Miyoshi H Kameda H Nomoto H. Usefulness of the octreotide test in Japanese patients for predicting the presence/absence of somatostatin receptor 2 expression in insulinomas. Endocr J. (2016) 63:135–42. 10.1507/endocrj.EJ15-0371 26567922

[15] Romeo S Milione M Gatti A Fallarino M Corleto V Morano S. Complete clinical remission and disappearance of liver metastases after treatment with somatostatin analogue in a 40-year-old woman with a malignant insulinoma positive for somatostatin receptors type 2. Horm Res. (2006) 65:120–5. 10.1159/000091408 16479142

[16] Oberg K Lamberts SW. Somatostatin analogues in acromegaly and gastroenteropancreatic neuroendocrine tumours: past, present and future. Endocr Relat Cancer. (2016) 23:R551–66. 10.1530/ERC-16-0151 27697899

[17] Yau H Kinaan M Quinn SL Moraitis AG. Octreotide long-acting repeatable in the treatment of neuroendocrine tumors: patient selection and perspectives. Biologics. (2017) 11:115–22. 10.2147/BTT.S108818 29255345 PMC5723116

[18] Pisarek H Stepien T Kubiak R Borkowska E Pawlikowski M. Expression of somatostatin receptor subtypes in human thyroid tumors: the immunohistochemical and molecular biology (RT-PCR) investigation. Thyroid Res. (2009) 2:1. 10.1186/1756-6614-2-1 19173713 PMC2646698

[19] Klagge A Krause K Schierle K Steinert F Dralle H Fuhrer D. Somatostatin receptor subtype expression in human thyroid tumours. Horm Metab Res. (2010) 42:237–40. 10.1055/s-0029-1243636 20094970

[20] Cheung NW Boyages SC. The thyroid gland in acromegaly: an ultrasonographic study. Clin Endocr. (1997) 46:545–9. 10.1046/j.1365-2265.1997.1680985.x 9231049

[21] Misaki T Maciel RM Tramontano D Moses AC Lombardi A Ingbar SH. Supranormal stimulation of deoxyribonucleic acid synthesis in FRTL5 cells by serum from patients with untreated acromegaly. J Clin Endocrinol Metab. (1988) 66:1227–32. 10.1210/jcem-66-6-1227 3372685

[22] Tode B Serio M Rotella CM Galli G Franceschelli F Tanini A. Insulin-like growth factor-I: autocrine secretion by human thyroid follicular cells in primary culture. J Clin Endocrinol Metab. (1989) 69:639–47. 10.1210/jcem-69-3-639 2547829

[23] Bogazzi F Manetti L Bartalena L Gasperi M Grasso L Cecconi E. Thyroid vascularity is increased in patients with active acromegaly. Clin Endocrinol. (2002) 57:65–70. 10.1046/j.1365-2265.2002.01562.x 12100071

[24] Roelfsema F Frolich M. Pulsatile thyrotropin release and thyroid function in acromegalics before and during subcutaneous octreotide infusion. J Clin Endocrinol Metab. (1991) 72:77–82. 10.1210/jcem-72-1-77 1986030

[25] Tonacchera M Agretti P Rosellini V Ceccarini G Perri A Zampolli M. Sporadic nonautoimmune congenital hyperthyroidism due to a strong activating mutation of the thyrotropin receptor gene. Thyroid. (2000) 10:859–63. 10.1089/thy.2000.10.859 11081252

[26] Murakami M Kamiya Y Yanagita Y Mori M. Gs alpha mutations in hyperfunctioning thyroid adenomas. Arch Med Res. (1999) 30:514–21. 10.1016/S0188-4409(99)00078-8 10714366

[27] Landis CA Masters SB Spada A Pace AM Bourne HR Vallar L. GTPase inhibiting mutations activate the alpha chain of Gs and stimulate adenylyl cyclase in human pituitary tumours. Nature. (1989) 340:692–6. 10.1038/340692a0 2549426

